# Multi-isocentric volumetric arc-based total body irradiation: radiation dose impact on oncological outcomes in patients receiving allogeneic hematopoietic cell transplantation

**DOI:** 10.3389/fonc.2025.1695178

**Published:** 2026-01-09

**Authors:** Anna Romanowska, Joanna Kamińska, Alicja Sadowska-Klasa, Anita Prawdzik–Dampc, Renata Zaucha

**Affiliations:** 1 Department of Oncology and Radiotherapy, University Clinical Center in Gdansk, Gdańsk, Poland; 2 Department of Oncology and Radiotherapy, Medical University of Gdansk, Gdańsk, Poland; 3 Department of Hematology and Transplantology, Medical University of Gdansk, Gdańsk, Poland

**Keywords:** bone marrow transplantation, conditioning, radiotherapy dose, total body irradiation dose, volumetric modulated arc therapy

## Abstract

**Background and purpose:**

Total body irradiation (TBI) is an important component of conditioning schedules. Data comparing different radiotherapy (RT) regimens in allogeneic hematopoietic cell transplantation (allo-HCT) remain limited. We aimed to evaluate the oncological outcomes of patients receiving different RT doses.

**Materials and methods:**

All patients treated with multi-isocentric volumetric arc-based total body irradiation (VMAT-TBI) at a dose of ≥8 Grays (Gy) between 2021 and 2023 were included in this retrospective analysis. The RT regimens were either 8 Gy delivered in 4 bi-daily fractions (fx) or 12 Gy in 6 bi-daily fx. We evaluated the overall survival (OS), relapse-free survival (RFS), engraftment, and toxicities in both groups.

**Results:**

Forty-two patients met the inclusion criteria, including 24 treated with a 12 Gy regimen. Hazard ratio for OS after adjusting for age, Charlson Comorbidity Index (CCI), Disease Risk Index (DRI), disease status and total conditioning score (TCI) for survival was 0.02 (0.00, 0.48. p=0.01) in favor of a higher dose. The median RFS in the 12 Gy cohort was not achieved, and it was 11.8 months in the 8 Gy group. Toxicities were comparable between the groups. Two treatment-related deaths occurred in the 8 Gy arm. All patients in the 12 Gy arm achieved engraftment, whereas one graft failure was observed in the 8 Gy arm.

**Conclusion:**

In patients receiving modern TBI before allo-HCT with high-quality dose distribution, 12 Gy appears to be more effective than 8 Gy. However, this concerns a single-center cohort with TBI dose allocation according to estimated patient fragility before transplant. Therefore, randomized trials are required to determine the optimal RT dose.

## 1 Introduction

First introduced in the late fifties, total body irradiation (TBI) remains a fundamental component of conditioning regimens before hematopoietic cell transplantation (HCT) for selected hematological malignancies and some benign diseases (1–3). TBI aims to eradicate tumor cells, especially those from sanctuary sites, and provides adequate bone marrow depletion and immunoablation together with chemotherapy to enable engraftment. Despite the standard use of TBI, with 12 Grays (Gy) delivered in six fractions (fx) bidaily being the most common schedule in the myeloablative approach, more data on the optimal radiotherapy dose need to be collected (4). Dose escalation usually result in increased toxicity without improving patient outcomes (5). Over the last two decades, the results of several trials and cohorts have shown that 8 Gy TBI is not inferior to the 12 Gy TBI dose (4, 6, 7). Recently, EBMT registry-based analyses suggested an 8 Gy TBI dose as the standard of care for patients with acute lymphoblastic leukemia (ALL) (8). Due to the contradictory results of prospective trials, we treated patients with either 12 Gy/6 fractions (fx)/3days standard dose TBI (sTBI) or a reduced-intensity radiotherapy regimen (rTBI) of 8 Gy/4 fx/2 days following the transplant qualifying committee decision. Using the volumetric arc-based irradiation (VMAT-TBI) technique implemented at our center in 2021, we achieved excellent dose homogeneity and proper protection of organs at risk (OARs). VMAT-TBI with rotating gantry, continuous shaping and varying the intensity of radiation beams allows to deliver precise, highly conformal doses to the whole body while significantly reducing radiation to OARs. Here, we present a retrospective analysis of these results. This is the first report on the impact of a homogeneously delivered TBI dose on allogeneic hematopoietic cell transplantation (allo-HCT) treatment outcomes.

## 2 Materials and methods

### 2.1 Patients

The study group consisted of all adult patients with hematological malignancies treated with VMAT-TBI at the dose ≥8 Gy in our institution between 2021 and 2023.

This study was conducted in accordance with the latest guidelines of the Declaration of Helsinki. All patients signed an informed consent form before participating in the transplantation procedures. For further analyses, approval was obtained from the hospital authorities of the University Clinical Center of Gdańsk and the Bioethical Committee at Medical University of Gdańsk (KB/495/2023).

### 2.2 Data collection and statistical analysis

Radiotherapy (RT) details, patient and disease characteristics, toxicities, engraftment data, and follow-up data were obtained from anonymized medical records. Toxicities were graded according to the Common Toxicity Criteria for Adverse Events (CTCAE) v. 5.0.

### 2.3 Statistical analysis

Overall survival (OS) was calculated from the allo-HCT date to the date of death or last follow-up. Relapse-free survival (RFS) was defined as the time interval between allo-HCT and disease recurrence. Survival probabilities over time were modeled using the Kaplan-Meier method, and differences between survival curves were assessed using the log-rank test. The Cox Proportional Hazards model was employed to analyze the association between the TBI dose and the risk of relapse or death. Factors showing significant differences between the TBI dose groups, as identified by the Mann–Whitney U test, were included as covariates in the Cox model. These included the Charlson Comorbidity Index (CCI) (9), Disease Risk Index (DRI) (10, 11), disease status (e.g., complete remission, minimal residual disease status, and active disease), patient age, and total conditioning score (TCI) (12). Given the limited sample size and number of events (13 deaths total), we acknowledge that our multivariable model with 5 covariates yields an events-per-variable (EPV) ratio of 2.6, which is below the generally recommended threshold of 10-15. Therefore, the multivariable results should be considered exploratory and interpreted with caution. All reported confidence intervals reflect this limitation, and findings require validation in larger cohorts. Analyses were performed using the Lifeline Survival Analysis Package in Python.

### 2.4 Conditioning regimens

Conditioning regimen with the use of TBI (TBI-Fludarabine or TBI-Cyclophosphamide) was a standard of care for all patients with ALL. Other indications included chronic lymphoproliferative diseases, acute myeloid leukemia (AML) with central nervous system involvement, or extramedullary disease. Patients transplanted from fully matched donors received cyclosporine and methotrexate as routine graft-versus-host prophylaxis, with the addition of antithymocyte globulin for unrelated donors. When a mismatched donor was used, posttransplantation cyclophosphamide with mycophenolate mofetil and tacrolimus was administered. The TBI dose was usually reduced in elderly patients (>60 years old), younger patients with significant comorbidities or poor general performance status, and patients qualified for a second or rescue transplantation. Peripheral blood was used as the stem cell source in all cases.

### 2.5 Radiotherapy

Patients were immobilized in a supine position on an individualized vacuum mattress (Orfit Industries Wijnegem, Belgium) at Orfit SBRT Based Plate™ (Orfit Industries Wijnegem, Belgium) using thermoplastic masks for head and knees immobilization. Two sets of computed tomography (CT) images, head-first (HFS) and feet-first (FFS), were obtained for each patient. The planning target volume (PTV) was defined as the whole body with a 3–4 mm contraction for most of the patients. The lungs and kidneys were contoured as the organs at risk (OARs). The RT dose was either 8 or 12 Gy. The schedule choice was determined by the hospital transplant team. RT was performed twice daily with a minimum interval of 6 h between fx. In some cases of persistent disease, an additional boost dose was administered pre-TBI. The planning goals were to deliver at least 90% of the planned dose (PD) to at least 95% of the PTV (V_90_>95%), limit the volume receiving >110% of the PD to less than 10% of the body volume (V_110_<10%), and achieve mean lung and kidney doses below 10 Gy (optimally 8 Gy) for patients receiving 12 Gy of treatment. RT was performed using VMAT. The dose was delivered on a TrueBeam Accelerator (Varian Medical System Inc., Palo Alto, USA) with 6 MV photon beams and pre-fraction kilovoltage orthogonal imaging for setup verification.

Finding a common reference point for both sets of CT scans is crucial for plan optimization. We used the upper edge of the long hole in the middle of the plate, originally designed for leg support fixation, as an additional orientation point. This allowed us to determine the positions of the lowest isocenter for the HFS and the uppermost isocenter for the FFS. The positions of the remaining isocenters were determined with reference to those two. The coronal markers were placed at an appropriate height on the table so that the gantry could perform a full arc rotation. Thus, all isocenters had the same anteroposterior coordinates. Ten isocenters—seven isocenters in the HFS position and three isocenters in the FFS position (6MV beams over the longitudinal axis of the head and legs and lateral axis for the chest, abdomen, and pelvis; 13 arcs and 18 half-arcs)—were generated and optimized for each patient to deliver a uniform dose. The distance between the isocenters was 20–25 cm. The maximum overlapping region between the fields was 3 cm.

The PTV was divided into three subsections: head and chest, abdomen and pelvis, and legs. OARs were subtracted from the PTV when required. All plans were optimized using a photon optimization algorithm, the Analytical Anisotropic Algorithm (AAA), in the Eclipse Treatment Planning System, version 16.1 (Varian Medical Systems Inc., Palo Alto, USA). The head and chest were optimized together, followed by the abdominal and pelvic beams. Finally, the calculated fields for the HFS orientation were added to the fields for the FFS orientation to perform the final optimization.

## 3 Results

Between 2021 and 2023, 42 patients were treated with VMAT-TBI with doses ≥ 8 Gy. In 24 cases, 12 Gy/6 fx/3 days was administered at 18 cases 8 Gy/4 fx/2 days. Six patients received boost doses for persistent disease at various locations. In the sTBI group, the boosted areas were the thoracic chest wall in two patients, the mediastinum in one, and the sacral nerve root in one patient. In the rTBI cohort, an additional dose was administered to the mediastinum and right hip. The most common diagnosis was acute lymphoblastic leukemia (ALL); however, in rTBI, more patients underwent TBI because of AML. The patient characteristics during the TBI procedure are summarized in **Table 1**. The median age of the patients was 34.5 years in sTBI vs 53.5 years in rTBI (range, 19–67). All patients had been pretreated with a median of one therapy line (range, 1–7). Most of the patients were transplanted in complete remission with a negative minimal residual disease (MRD) status (56% in rTBI, 88% in sTBI). Active disease was observed more frequently in the rTBI group (33%) than in the sTBI group (8%). Eight patients had extramedullary disease (**Table 1**).

**Table 1:** Patients characteristics.

Characteristics	rTBI (n=18)	sTBI (n=24)	p
Age	53.5 (32-67)	34.5 (19-52)	(p<0.0001)
Diagnosis	ALL n=6 AML n=7 other n=5	ALL n=17 AML n=3 other n=4	p=0.04 (chi^2)
Disease status before Tx	CR, MRD (-) n=9 CR, MRD (+) n=2 sCR n=1 active n=6	CR, MRD (-) n=17 CR, MRD (+) n=4 sCR n=1 active n=2	p=0.2 (chi^2)
Donor	MUD n=9 MRD n=7 mMRD n=1 mMUD n=1	MUD n=14 MRD n=5 mMRD n=4 mMUD n=1	p=0.5 (chi^2)
Conditioning	Flu150 n=15 Other n=3	Flu150 n=18 Cy120 n=6	p=0.8 (chi^2)
Intrathecal treatment	n =6	n=19	p=0.007 (chi^2)
Previous chemotherapy lines	2 (1-7)	1 (1-3)	p=0.05
TCI score	2.5 (2.5-3.5)	3.5 (3.5-4)	p<0.00005
CCI	3.5 (2-9)	2 (2-5)	p=0.01
DRI	Low n=1 Intermediate n=10 High n=4 Very high n=3	Low n=0 Intermediate n=18 High n=3 Very high n=3	p=0.5 (chi^2)

ALL, Acute Lymphocytic Leukemia; AML, Acute Myeloblastic Leukemia; CCI, Charlson Comorbidity Index; CR, Complete Remission; DRI, Disease Related Index; mMRD, mismatched related donor; mMUD, mismatched unrelated donor; MRD, minimal residual disease or matched related donor; MUD, matched unrelated donor; TCI, Total Conditioning Index; rTBI, reduced dose TBI; sTBI, standard dose TBI.

**Figure 1:**
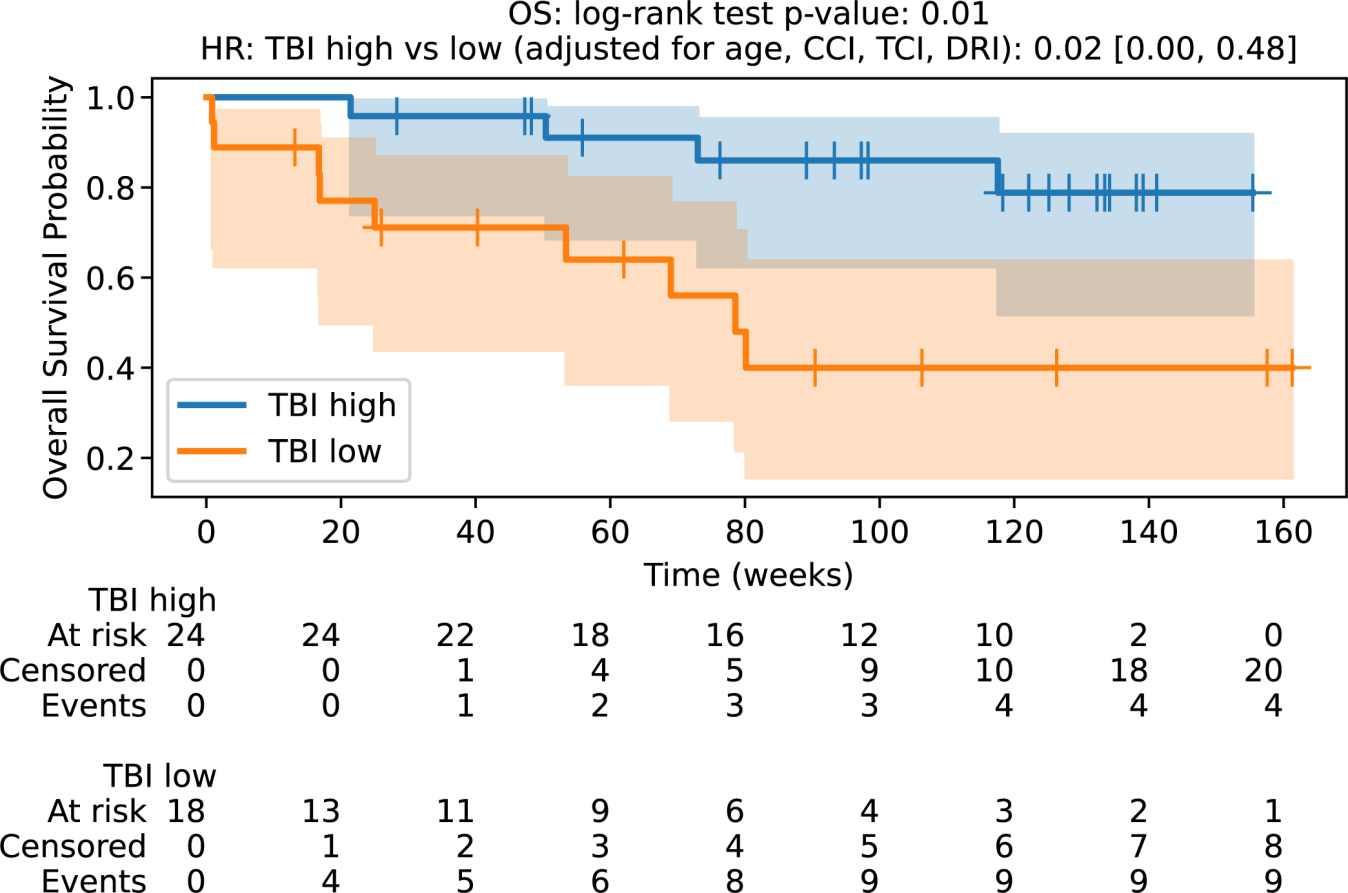
Overall survival probability. OS, overall survival; TBI Iow, reduced dose TBI 8Gy/4fx/2days; TBI high, standard dose TBI 12Gy/6fx/3days.

**Figure 2:**
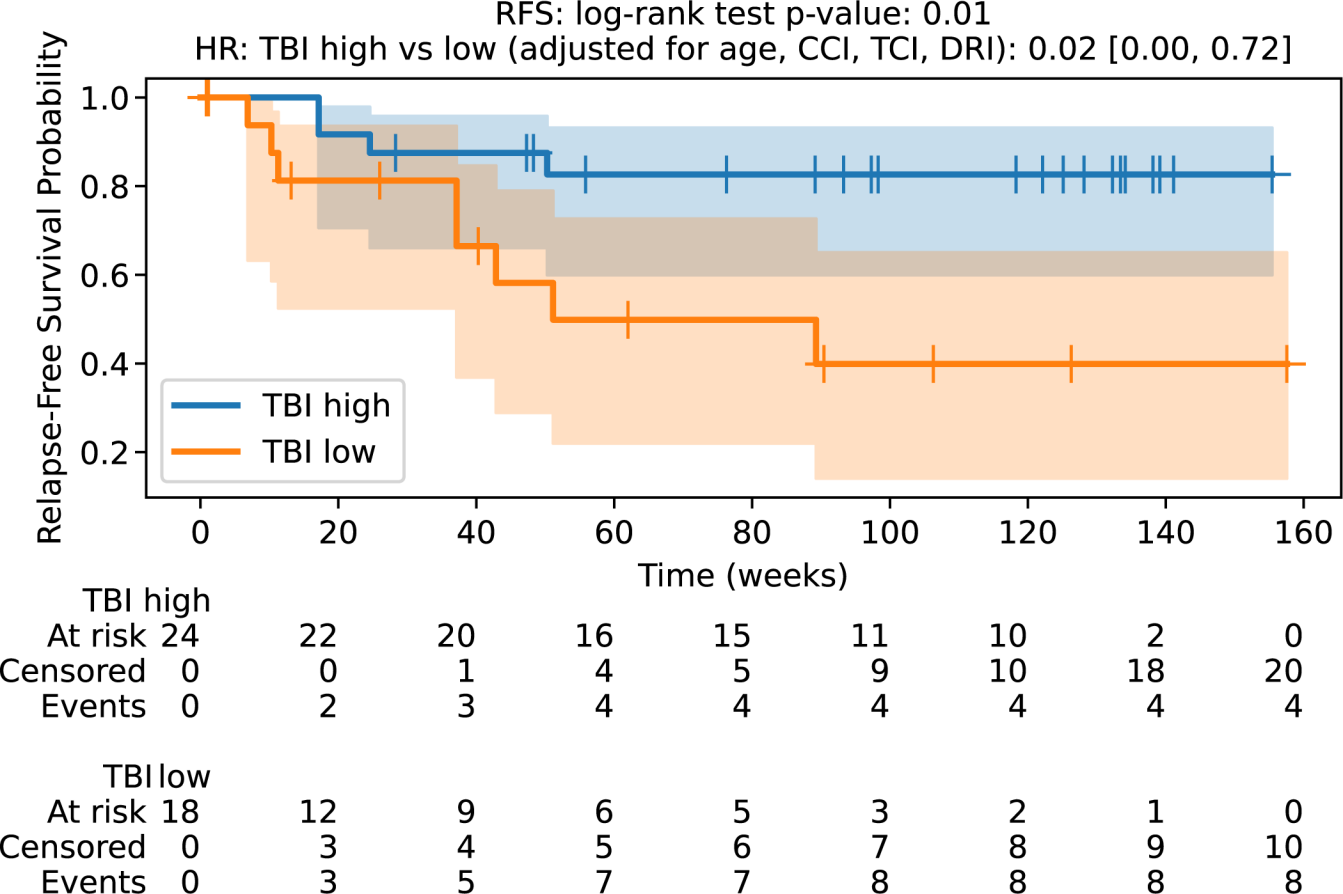
Relapse-free survival probability. RFS, relapse-free survival; TBI Iow, reduced dose TBI 8Gy/4fx/2days; TBI high, standard dose TBI 12Gy/6fx/3days.

The median OS in the 8 Gy cohort was 18.1 months, which was not achieved in the 12 Gy group with a median follow-up of 28.1 months. The hazard ratio for survival after adjusting for age, CCI, TCI, and DRI was 0.02 (0.00, 0.48) with p-value of 0.01 (**Figure 1**). The median RFS in the sTBI cohort was not achieved, and it was 11.8 months in the rTBI group. The hazard ratio (HR) for RFS was 0.02 (0.00, 0.72), with a p-value of 0.01 (**Figure 2**). The two-year OS rates in the sTBI and rTBI groups were 83% and 40%, respectively. Nine patients in the rTBI arm died, including six from disease relapse. In the sTBI arm, four patients died from relapse, without treatment-related mortality. Statistical details of multivariate analysis are summarized in **Supplementary Tables S1, S2** in supplementary files.

Toxicities were acceptable and comparable between the groups. Acute mucositis was higher in the sTBI group. Two treatment-related deaths occurred in the rTBI cohort: one from pneumonia leading to multi-organ failure and one from sepsis with multi-organ failure. None occurred in the sTBI group. Late toxicities were mostly mild and similar between the cohorts, including the patients boosted to the areas of the persistent disease. There was one death due to sepsis in the rTBI group. Endocrine disorders developed in about 1/3 of the patients and included 4 cases of hypothyroidism, one adrenal insufficiency and premature menopause in 8 women. Differences between groups were statistically insignificant. The details are presented in **Table 2**.

**Table 2:** Toxicity details.

Toxicity Group	CTCAE v 5.0 Grade	rTBI (18) Number (Percent) of Patients	sTBI (24) Number (Percent) of Patients	p
Acute toxicities				
Skin	G1	2 (11%)	1 (4%)	p=0.7 (chi^2)
	G2	0	1 (4%)	
Nausea and vomiting	G1	5 (28%)	4 (17%)	p=0.2 (chi^2)
	G2	0	3 (12%)	
	G3	1 (6%)	0	
Gastro-intestinal	G1	3 (17%)	7 (29%)	p=0.5 (chi^2)
	G2	3 (17%)	4 (17%)	
	G3	1 (6%)	3 (12%)	
	G4	0	1 (4%)	
Lung	G1	1 (6%)	1 (4%)	p=0.8 (chi^2)
	G2	2 (11%)	2 (8%)	
	G3	1 (6%)	2 (8%)	
	G4	0	0	
	G5	1 (6%)	0	
Mucositis	G1	3 (17%)	0	p=0.02 (chi^2)
	G2	2 (11%)	3 (12%)	
	G3	5 (28%)	11 (46%)	
	G4	4 (22%)	10 (42%)	
Other	G1	5 (28%)	10 (42%)	p=0.4 (chi^2)
	G2	2 (11%)	5 (21%)	
	G3	2 (11%)	1 (4%)	
	G4	0	1 (4%)	
	G5	2 (11%)	0	
Late toxicities				
Lung	G1	3 (17%)	2 (8%)	p=0.7 (chi^2)
Kidney	G1	2 (11%)	1 (4%)	p=0.8 (chi^2)
Endocrine	G2	4 (22%)	8 (33%)	p=0.7 (chi^2)
Other	G2	1 (6%)	2 (8%)	p=0.7 (chi^2)
	G3	1 (6%)	1 (4%)	
	G5	1 (6%)	0	

CTCAE, Common Toxicity Criteria for Adverse Events; G1-5, grade in CTCAE; rTBI, reduced dose TBI 8Gy/4fx/2days; sTBI, standard dose TBI 12Gy/6fx/3days.

In the sTBI group, all patients achieved engraftment defined as an absolute neutrophil count >0.5 G/l in 3 consecutive days. The rTBI group had one graft failure and two early deaths before hematopoiesis was restored. The median time until sustained neutrophil engraftment and platelet counts of more than 50 G/l was adequately 21.5 (range, 14–36) and 22 days (range, 12–40) in the rTBI group and 23 (range, 13–42) and 26.5 (range, 14–52) days in the sTBI group.

## 4 Discussion

For many years, TBI has been an important component of conditioning regimens, especially for ALL. Destroying neoplastic cells throughout the body independent of blood distribution gives it the privilege to reach the sanctuary sites of the disease (13). TBI-containing conditioning regimens also have advantages in terms of OS and risk of relapse (14, 15). Despite its long clinical history, the optimal radiotherapy dose and scheduling remain unknown, with doses of 8–14 Gy commonly used in routine practice (16, 17). Historically, fractionated TBI with doses <10 Gy resulted in worse engraftment and a higher relapse rate compared to doses >12 Gy, which were associated with increased non-relapse mortality and a higher incidence of late sequelae (5, 18–23). Hence, 12 Gy in bi-daily fractions has become the most frequent regimen, although it has not been developed systematically in dose escalation studies (4, 24). We identified only two randomized trials that compared different radiotherapy doses. The first study published in 1990 compared the doses of 12 and 15.75 Gy in patients with AML in first remission. The trial showed the advantage of a higher dose in terms of relapse rate but did not improve survival because of increased treatment-related mortality (5). Second, to address the same population, TBI doses of 8 Gy and 12 Gy were compared. The trial recruited 195 patients and was terminated earlier because of poor accrual. This study showed comparable oncological outcomes with lower toxicity in the 8 Gy arm (4). In most studies that demonstrated the benefit of adding TBI to conditioning regimens, a high dose was used (15, 25, 26). Therefore, most treatment guidelines recommend doses ≥12 Gy (13, 17, 24, 27). The basics of using reduced-intensity conditioning TBI-containing regimens come mainly from retrospective, registry-based analyses or one-arm series (6, 7, 28). The rationale for dose reduction is to lower transplant-related mortality, focusing more on graft-versus-tumor effects than on myeloablation. Thus, the risk of relapse is higher (29, 30). In most trials, radiotherapy details were either unknown or the technique used was old; thus, dose distribution in the body and organs at risk can significantly differ from the prescribed ones. The technique of TBI delivery can affect both safety and efficacy and varies among centers (16, 17). Several reports have suggested improved long-term toxicity outcomes with modern TBI delivery techniques (31). In a recent EBMT-based analysis, the authors showed that rTBI was not inferior to sTBI in patients with ALL in their first complete remission. However, radiotherapy data in the review are scarce, with an unknown radiotherapy schedule for 28% of the patients and a lack of technical details (8).

In our study, all patients received VMAT-TBI, with three-dimensional dose evaluation ensuring planned target coverage and organ protection. sTBI showed superior OS and RFS in multivariate analysis. However, the rTBI group included more elderly patients with active or resistant/refractory disease and a higher CCI. Mucositis grade and gastrointestinal toxicities were higher in the sTBI group; however, no treatment-related deaths or excessive late toxicities occurred.

This study has several important limitations that warrant careful consideration when interpreting our findings. First, the retrospective design inherently limits causal inference, as treatment allocation was not randomized. The decision to use 8 Gy versus 12 Gy TBI was made by our institutional transplant committee based on patient-specific factors including age, comorbidities, and performance status. This creates substantial baseline differences between cohorts: the rTBI group was significantly older, had higher comorbidity burden, different disease distribution, and higher proportion of active disease. While we employed multivariable Cox regression to adjust for these differences, such adjustment cannot completely eliminate selection bias or account for unmeasured confounding factors.; However, it is the first study to evaluate TBI dose in the modern radiotherapy era, with full radiotherapy data available and uniform radiation technique and delivery among all participants. The main imbalance between groups was the diagnosis, with more AML patients in rTBI group. To minimize this bias we used DRI, which correlates with patient’s prognosis, as one of covariates when calculating HR for OS and RFS.

We believe that strong data is lacking for the routine use of a reduced TBI dose in myeloablative conditioning for standard indications, especially in younger populations with a high-risk disease. In patients with a poor performance status or advanced age, reduced-intensity regimens with or without TBI can be considered to avoid treatment-related deaths.

Patients with resistant or active disease can benefit from 12 Gy TBI with a local dose reduction to prevent critical organ damage because the relapse rate in this population was significantly higher after the reduced conditioning regimen.

The optimal radiotherapy dose may differ in individual patients. Those with higher risk disease can potentially benefit from dose escalation. Increasing the dose usually results in lower relapse rate but higher non relapse mortality and doesn’t improve patient’s survival. Additionally the heterogeneity in not only total dose, but fractionation and setup technique may hamper proper comparisons on a large multicenter scale (32, 33).Lately some promising attempts of more targeted radiotherapy focusing on the skeleton or even on active bone marrow were reported, but their implementation into routine practice requires further research (34, 35).We believe that the future of TBI is in individualizing the therapy also with VMAT technique to minimize side effects in order to take the full advantage from the disease-controlling potential of irradiation.

## 5 Conclusions

The VMAT-TBI dose appeared to be a relevant factor influencing patient outcomes. Fear of excessive treatment toxicity should not be the reason for RT dose reduction. We believe that a dose of 12 Gy should be maintained for all eligible patients to overcome the toxicity limitations in personalized dose modulation with novel RT techniques. Further research and randomized studies are needed to determine the optimal radiotherapy schedule.

## Data Availability

The datasets presented in this article are not readily available because Research data are stored in an institutional repository and will be shared upon request to the corresponding author after institutional approval. Requests to access the datasets should be directed to Anna Romanowska, aromanowska@uck.gda.pl.
