# Supplementary material for: Multi-isocentric volumetric arc-based total body irradiation: radiation dose impact on oncological outcomes in patients receiving allogeneic hematopoietic cell transplantation
Source: Front Oncol. 2026 Jan 9;15:1695178. doi: 10.3389/fonc.2025.1695178 (PMC12827096; doi:10.3389/fonc.2025.1695178)
Supplement: Supplementary file 1 [file Table1.docx]

**Table 1S.** Multivariate analysis for Overall Survival.

| covariate | coef | exp  (coef) | se  (coef) | coef lower 95% | coef upper 95% | Exp  (coef)  Lower  95% | Exp  (coef) upper 95% | z | p | -log2(p) |
| --- | --- | --- | --- | --- | --- | --- | --- | --- | --- | --- |
| RT dose | -3.838 | 0.021 | 1.581 | -6.937 | -0.738 | 0.001 | 0.478 | -2.427 | 0.015 | 6.036 |
| Age | 0.0454 | 1.046 | 0.036 | -0.025 | 0.116 | 0.975 | 1.123 | 1.261 | 0.207 | 2.271 |
| TCI | 2.409 | 11.123 | 1.198 | 0.061 | 4.757 | 1.063 | 116.373 | 2.011 | 0.044 | 4.496 |
| CCI | -0.269 | 0.764 | 0.355 | -0.966 | 0.426 | 0.380 | 1.531 | -0.760 | 0.440 | 1.160 |
| DRI | 0.619 | 1.856 | 0.350 | -0.068 | 1.305 | 0.934 | 3.690 | 1.765 | 0.077 | 3.687 |

**Table 2S.** Multivariate analysis for Relapse Free Survival.

| covariate | coef | exp  (coef) | se  (coef) | coef lower 95% | coef upper 95% | Exp  (coef)  Lower  95% | Exp  (coef) upper 95% | z | p | -log2(p) |
| --- | --- | --- | --- | --- | --- | --- | --- | --- | --- | --- |
| RT dose | -4.103 | 0.0165 | 1.927 | -7.881 | -0.326 | 0.001 | 0.722 | -2.129 | 0.033 | 4.910 |
| Age | 0.058 | 1.060 | 0.0399 | -0.020 | 0.137 | 0.980 | 1.146 | 1.465 | 0.143 | 2.808 |
| TCI | 2.638 | 13.986 | 1.500 | -0.303 | 5.579 | 0.738 | 264.922 | 1.758 | 0.079 | 3.666 |
| CCI | -0.712 | 0.490 | 0.428 | -1.551 | 0.127 | 0.212 | 1.135 | -1.664 | 0.096 | 3.380 |
| DRI | 0.682 | 1.978 | 0.376 | -0.056 | 1.419 | 0.946 | 4.135 | 1.812 | 0.070 | 3.838 |

Legend: CCI – Charlson Comorbidity Index, DRI – Disease Related Index, TCI – Total Conditioning Index, RT dose – radiotherapy dose (rTBI versus sTBI), coef - the estimated coefficient for the predictor variable (log hazard ratio), exp(coef): the exponentiated coefficient, also known as the hazard ratio, se(coef) - the standard error of the coefficient estimate, coef lower 95% and coef upper 95% - he lower and upper bounds of the 95% confidence interval for the coefficient, exp(coef) lower 95% and exp(coef) upper 95% - the lower and upper bounds of the 95% confidence interval for the hazard ratio, z: z-score for the Wald test of the null hypothesis that the coefficient is zero, p - p-value corresponding to the z-test, representing the probability of observing the data if the true coefficient were zero, -log2(p) - the negative log base 2 of the p-value

Początek formularza

Dół formularza
